# Frequency of JAK2 V617F mutation in patients with Philadelphia positive Chronic Myeloid Leukemia in Pakistan

**DOI:** 10.12669/pjms.301.3906

**Published:** 2014

**Authors:** Najia Tabassum, Mohammed Saboor, Rubina Ghani, Moinuddin Moinuddin

**Affiliations:** 1 Najia Tabassum, Baqai Institute of Heamatology, Baqai Medical University, Karachi, Pakistan.; 2 Mohammed Saboor, Baqai Institute of Heamatology, Baqai Medical University, Karachi, Pakistan.; 3 Rubina Ghani, Department of Biochemistry, Baqai Institute of Heamatology, Baqai Medical University, Karachi, Pakistan.; 4 Moinuddin Moinuddin, Baqai Institute of Heamatology, Baqai Medical University, Karachi, Pakistan.

**Keywords:** Chronic myeloid leukemia, Philadelphia chromosome, BCR-ABL, JAK2 V617F mutation, JAK-STAT pathway

## Abstract

***Background and Objective:*** Co-existence of myeloproliferative disorders (MPD) and Janus associated kinase 2 mutation (JAK2 V617F) is a well-established fact. Only few case reports are available showing presence of JAK2 V617F mutation in chronic myeloid leukemia (CML). Purpose of this study was to determine the frequency of JAK2 V617F mutation in Philadelphia Chromosome positive (Ph ^+^) CML patients in Pakistan.

***Methods: ***The study was conducted from August 2009 to July 2010 at Civil Hospital and Baqai Institute of Hematology (BIH) Karachi. Blood samples from 25 patients with CML were collected. Multiplex reverse transcription polymerase chain reaction (RT-PCR) was performed for Breakpoint Cluster Region – Abelson (BCR-ABL) rearrangement. Conventional PCR was performed for JAK2 V617F mutation on BCR-ABL positive samples.

***Results:*** All 25 samples showed BCR-ABL rearrangement. Out of these 11 samples (44%) had JAK2 V617F mutation; the remaining 14 (56%) cases showed JAK2 617V wild type.

***Conclusion:*** It is concluded that the co-existence of Ph ^+^CML and JAK2 V617F mutation is possible.

## INTRODUCTION

The pathognomonic marker of CML is Ph chromosome that results from a reciprocal chromosomal translocation between the ABL gene on chromosome 9 with BCR gene on chromosome 22 t (9;22). The resultant chimeric oncogene BCR-ABL displays elevated tyrosine kinase (TYK) activity.^1^ Their cellular effect is exerted through activation of multiple signal transduction pathways that transduce oncogenic signals.^2^

- JAK2 belongs to the family of intracellular non-receptor TYK.ITITI It has seven Janus homology (JH) domains (JH1-JH7).^3^ JH1 is an elevated tyrosine kinase domain whereas JH2 is an inactive pseudokinase domain. JH2 has auto inhibitory properties therefore any alteration leads to constitutive tyrosine phosphorylation.^4^^,^^5^ Guanine is present on both loci (G/G) at codon 617 of JAK2.^6^ There are three types of JAK2 V617F mutation. Homozygous; alleles are mutant (T/T), heterozygous; one allele is wild and the other is mutant (G/T) and hemizygous where one allele is mutant and the other is absent (T/-).^7^ Mutant allele indicates mutation of wild or normal allele.^8^
- JAK2 V617F is a somatic mutation where Guanine to Thymine (GT) point mutation at nucleotide 1849 occurs.^9^^,^^10^ Consequently substitution of valine (V) by phenylalanine (F) at codon 617 (V617F) within JH2 domain takes place.^11^
- JAK2 role in haematopoieses is expression of hematopoietic growth factors receptors on the cell surface. These receptors transmit erythropoietin (EPO), thrombopoietin (TPO), cytokines, growth factors e.g. 1L-3, IL-5 and Granulocyte-Monocyte colony stimulating factor (GM-CSF).^12^

JAK2 V617F is a gain of function mutation. It disrupts the auto-inhibitory property of JH2 leading to constitutive tyrosine kinase activation of JH1.^13^ There is constant activation of signal transducer and activation of transcription3 (STAT3), up regulation of anti-apoptotic protein Bcl-x_L_^3^ and enhanced AKT activity.^7^ This deregulated signaling induces clonal expansion of haematopoietic progenitors that are independent of normal growth factor control.^12^

## METHODS

This is an observational cross sectional study. It was conducted from August 2009 to July 2010 at BIH and Civil hospital, Karachi. Inclusion criteria were:

1. Newly diagnosed as well as previously treated CML patients on Hydroxyurea in chronic or accelerated phase.
2. Presence of Ph chromosome or BCR-ABL rearrangement.
3. Age ≥18yrs of either sex.

Exclusion criteria were

1. BCR-ABL negative CML.
2. History of any MPD such as polycythemia vera (PV), essential thrombocythemia (ET) and idiopathic myelofibrosis (IMF).
3. Patients treated with tyrosine kinase inhibitor.

A total number of 25 patients with CML presented during the above mentioned period fulfilled the inclusion criteria. Written consent was taken. The study was approved by ethical committee of BMU. Data were recorded on case report forms. Age, gender, first and last complete blood counts (CBC), bone marrow biopsy reports were recorded. Whole blood samples (10cc each) were collected in EDTA (Ethylene diamine tetra acetic acid) tubes. Each sample was divided into two aliquots and placed in two separate tubes. First tube had whole blood while the second tube had plasma.

For the collection of data for CML and JAK2 V617F mutation, the following hematological and molecular analyses were performed.

1. CBC and morphology of the blood smears.
2. BCR-ABL determination by RT- PCR using Seeplex kit, Korea.
3. JAK2 V617F mutation determination with break points by done by Conventional PCR using Seeplex kit from Korea.

Results of PCR were interpreted as shown in table-I upon comparison with control markers (M) provided with the Seeplex kit.

***Statistical analysis:*** Statistical package for social sciences (SPSS) version 16 was used for data analysis. Descriptive statistics was applied for calculating the frequency. 

## RESULTS

**Fig.1:**
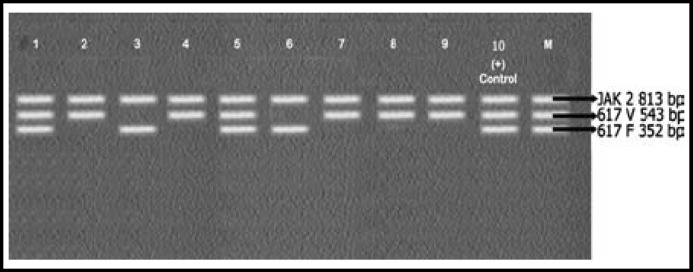
JAK2 Analysis of Samples 1 - 9

**Fig.2:**
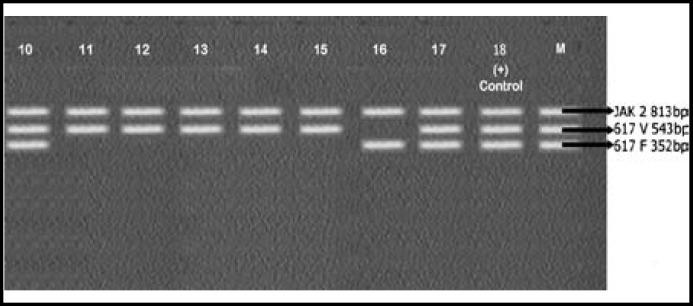
JAK2 analysis of Samples 10 – 17

**Fig.3:**
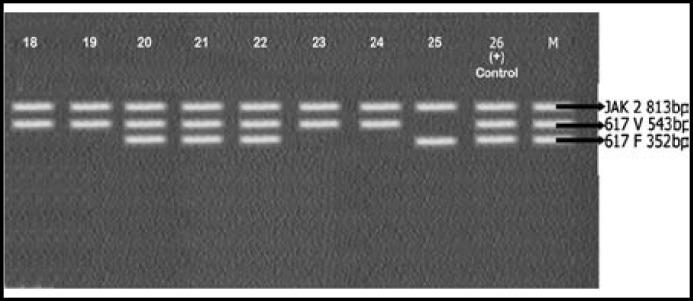
JAK2 analysis of Samples 18 – 25

A total of 25 (male 10, female 15) patients were enrolled in this study. Mean age of the patients was 51±2.5 years. CBC of patients showed increased leukocyte count with complete left shift. Bone marrow findings were consistent with that of CML. All 25 samples were positive for BCR-ABL rearrangements. Only 11 out of 25 (44%) samples were positive for JAK2 v v vvV617F mutation. The rest 14/25 (56%) showed JAK2 617V wild type. Results are shown in Fig. 1, 2, 3 and Table-II.

## DISCUSSION

Several studies have found close association between JAK2 V617F mutation and classic BCR-ABL negative MPD encompassing PV, ET and IMF. Over 95% of patients with PV and more than 50% of patients with ET and IMF harbor this mutation.^11^^,^^14^^-^^17^

Since Jelinek et al^10^ reported the absence of JAK2V617F mutation in patients with Ph^+^CML; it was thought that JAK2 V617F mutation and BCR-ABL translocation were mutually exclusive. However, Kramer et al.^18^ identified this mutation in a patient with Ph^+ ^CML and since then few similar cases has been reported.^19^^-^^25^ Out of these, Boochia et al.^19^ and Bee et al.^21^ patients with Ph^+^CML had a prior history of PV whereas Jalledes et al^22^ and Curtin et al^23^ reported cases had pre-existing JAK2 V617F positive ET who later acquired Ph translocation. Only cases of Nadali F et al.^24^ and Fava et al.^25^ had Ph +CML with concomittent JAK2 V617F mutation with no history of MPD.

**Table-I:** Interpretation of JAK2 results

*Condition*	*JAK2*	*617V*	*617F*	*Interpretation*
1	+	+	-	Not detected JAK2 V617F mutation. Specimen is wild type.
2	+	+	+	Detected JAK2 V617F mutation. Specimen is mutant type.
3	+	-	+	Detected JAK2V 617F mutation. Specimen is mutant type.

**Table-II:** Frequency of expression of JAK2V617F (mutant type) and JAK2617V (wild type).

*JAK2 Types*	*Number of cases (n=25)*	*% Frequency *
617F (mutant)	11	44
617V (wild)	14	56

Lane M is the marker while lane 10 is the positive control incorporated in the kit. Lane 1, 3, 5 and 6 shows JAK2 V617F mutation having an amplified product of 352 bp. Lane 2,4,7,8 and 9 shows JAK2617V (wild) having an amplified product of 543 bp.

Lane M is the marker while lane 18 is the positive control incorporated in the kit. Lane 10, 16 and 17 shows JAK2 V617F mutation having an amplified product of 352 bp. Lane 11,12,13,14 and 15 is JAK2617V (wild) having an amplified product of 543 bp.

Pahore et al.^20^ were the first to report the frequency of JAK2 V617F mutation in Pakistani patients with Ph^+^CML. In their study 26.7% of patients with CML carried this mutation. However, in our study 44% of the patients had this genetic aberration (Table-II). None of the patients in either study had pre-existing MPD. This is the second time that the frequency of this mutation is reported in Pakistani population. So far, this kind of study is not available in international literature.

The question is what is the role of JAK2 V617F mutation in Ph^+^ CML and how this combination can affect the pathogenesis, course of disease and its prognosis. Till now no definite answer has been sought. It is assumed that the presence of this mutation in Ph^+^CML may be behind the resistance to tyrosine kinase inhibitors.^25^

Hence it is concluded that the presence of JAK2 V617F mutation in almost half of the patients with Ph^+^CML in our study shows a possibility of the co-existence of these two disease specific mutations. Further studies on a larger scale are recommended to determine the exact frequency of JAK2 V617F mutation in Ph^+^CML. In case of persistent splenic enlargement or unexpected hematologic response during effective treatment of Ph^+^CML the possibility of an underlying JAK2 positive hematopoietic clone should always be entertained.
